# In memory of Marcos Vidal (1974-2016)

**DOI:** 10.1242/dmm.024612

**Published:** 2016-03-01

**Authors:** Ross Cagan, Eyal Gottlieb

**Affiliations:** 1 Icahn School of Medicine at Mount Sinai, Annenberg 25-40, Campus Box 1020, 1468 Madison Avenue, New York, NY 10029, USA; 2 Cancer Research UK Beatson Institute, Garscube Estate, Switchback Road, Bearsden, Glasgow, G61 1BD, UK

## Abstract

With the untimely death of Marcos Vidal, we have lost a good friend and a creative, brilliant colleague who made important contributions to the field of cancer biology through fruit fly research. Marcos began his research into *Drosophila* at Ross Cagan's laboratory in 2003, first at Washington University in St Louis and later at Mount Sinai Hospital in New York. In 2009 Marcos was appointed as Research Group Leader at the Beatson Institute for Cancer Research in Glasgow.


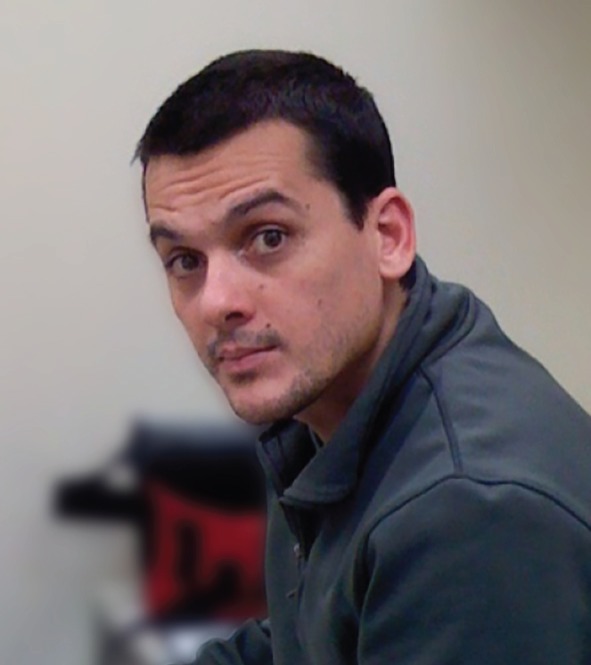


I (Ross) had just finished giving a talk in the Renal Division at Washington University School of Medicine when a young scientist approached me. He was a biochemist but liked the idea of using fruit flies to study diseases. Marcos Vidal was an impressive young man and I was excited to have him join the laboratory. A few days later a woman approached with the same request. Julia Cordero assured me that her relationship with Marcos would not be a problem, that they were used to working together and that they *never* fought. I sat them on opposite sides of the laboratory.

Marcos and Julia were a brilliant young couple who instantly made the laboratory smart and fun. Marcos took up cancer research, the second person to ever work on this disease in the laboratory. He loved everything about conducting science and he was ambitious. He worked crazy hours. With a laconic Argentinian accent, he kept up a running science conversation that lasted until now. Julia worked on separate topics and they lived balanced lives. Marcos loved board sports from snowboarding to kite- and wind-surfing, and was superb at them. Marcos and Julia made friends easily. The Facebook postings and the emails we've received and the conversations we've had over the past few days are a reminder of just how much they are loved. We all rooted for their success because they were really good people.

Marcos pushed the fly as a discovery platform further than almost anybody else. He was enthusiastic to exploit the power of drug screening. He had a feel for the organism and, perhaps most importantly, he had a feel for what constituted an important biological question. In 2005 he validated Vandetanib as a candidate therapeutic for medullary thyroid carcinoma. The drug was approved in 2011 for clinical use. He explored the role of Src in oncogenesis, providing evidence and mechanisms that position metastasis as an immediate early event, promoted at least in part by local cell interactions within the epithelium. Later on, as an independent Group Leader in Glasgow, Marcos focused on the cancer-promoting role of the tumor microenvironment and the immune system. Using the fruit fly as a cancer model, Marcos demonstrated that the genetic composition of a tumor is a key determinant as to whether tumor necrosis factor (TNF) acts as a tumor-suppressing or tumor-promoting factor. He further characterized the crosstalk between epithelial tumors and the innate immune response, demonstrating that the TNF-Toll non-cell-autonomous signaling cascade dictates tumor cell fate. Marcos used the fruit fly cancer model creatively, and he did it with a style unmistakably his own.

As a couple, Marcos and Julia were a true science power: young and smart and handsome. In Glasgow, Marcos started out as a Junior Group Leader at the Beatson Institute, becoming my (Eyal) valued neighbor and peer. Julia helped him set up his laboratory, and later started her own. They lived the lives of young professionals, with an international twist. They worried about keeping two leadership research positions, worried about their two young children and whether Argentinians could adapt to the Scottish weather. But they soon became central to the Beatson Institute, got involved with the local community and established successful professional and personal lives in Scotland. Beyond his work in the lab, Marcos dedicated his energy and free time to his family. He spent long, happy hours passing his boarding and drawing skills to his son Lautaro, and riding his bike with his daughter Mara.

Marcos fell ill 18 months ago and those extremely difficult times were a testimony to the central role his family and friends played in his life. Marcos worked as hard as he could to make a recovery. He received tremendous support from his colleagues and friends and, most of all, from Julia. Despite struggling with his illness, Marcos sailed through his tenure review with flying colors and, in July 2015, he became a Senior Research Group Leader at the Beatson Institute and a Professor at the University of Glasgow.

Marcos Vidal died on January 2, 2016. Julia, Lautaro and Mara lost a warm-hearted, dedicated and loving husband and father; the science community has lost a brilliant young scientist and a good friend.

